# *Dryopteriswulingshanensis* (Dryopteridaceae), a new species from Hunan, China

**DOI:** 10.3897/phytokeys.185.72019

**Published:** 2021-11-10

**Authors:** Jiang-Ping Shu, Zi-Yue Liu*, Zhi-Rong Gu, Li-Jun Chen, Hong-Jin Wei, Xi-Le Zhou, Yue-Hong Yan, Rui-Jiang Wang

**Affiliations:** 1 Key laboratory of Plant Resources Conservation and Sustainable Utilization, South China Botanical Garden, Chinese Academy of Sciences, Guangzhou, 510650, China; 2 Key Laboratory of National Forestry and Grassland Administration for Orchid Conservation and Utilization, Shenzhen, 518114, China; 3 Shenzhen Key Laboratory for Orchid Conservation and Utilization, The National Orchid Conservation Centre of China and The Orchid Conservation and Research Centre of Shenzhen, Shenzhen, 518114, China; 4 University of Chinese Academy of Sciences, Beijing, 100049, China; 5 National Engineering Research Center of Navel Orange, Gannan Normal University, Ganzhou, 341000, China; 6 Badagongshan National Nature Reserve, Hunan, 427100, China; 7 Shanghai Chenshan Botanical Garden, Shanghai, 201602, China; 8 Xiangxi Tujia and Miao Autonomous Prefecture Forest Resources Monitoring Center, Jishou 416000, China

**Keywords:** New taxon, *rbc*L, subg. *Dryopteris*, phylogeny, fern

## Abstract

*Dryopteriswulingshanensis*, a new species growing on limestone in the Wulingshan Mountains, Hunan, China, is described and illustrated. This species is most similar to *D.jishouensis* and *D.gymnophylla* on general morphological traits, such as the form of scales, rhizome and sori, but differs by the number of vascular bundles at the base of the petiole, length to width ratio of lamina, stalk length of basal pinnae, division of the lamina, apex form of the pinnule and habitat. Moreover, molecular phylogenetic analysis using the chloroplast *rbc*L gene suggested that *D.wulingshanensis*, as the sister group of *D.jishouensis*, is a monophyletic clade. According to its restricted geographic range, small populations and few individuals, *D.wulingshanensis* should be considered endangered, according to the IUCN Red List criteria.

## Introduction

*Dryopteris* Adans. (1763: 20, 551) is one of the largest fern genera with about 400 species, widely distributed all over the world (Wu et al. 2013). Based on molecular phylogenetic evidence, several genera are nested within *Dryopteris*, such as *Acrophorus* C. Presl, *Acrorumohra* (H. Itô) H. Itô, *Diacalpe* Blume, *Dryopsis* Holttum & P. J. Edwards, *Nothoperanema* (Tagawa) Ching and *Peranema* D. Don (Zhang and Zhang 2012; Zhang et al. 2012). Most species in *Dryopteris* share a short rhizome and catadromous arrangement of frond segments, compared to its sister genus, *Arachniodes* Blume, which has long-creeping rhizomes and anadromous laminae (Zhang et al. 2012; Wu et al. 2013). The species of this genus usually grow in forests, open vegetation and, occasionally, in the rocky area of temperate and tropical regions (Fraser-Jenkins 1986; Kramer et al. 1990; Wu et al. 2013). In China, the genus is widely distributed, especially in south-western regions, with about 167 species with 60 endemic species in four subgenera (D.subg. Pycnopteris, D.subg. Nothoperanema, D.subg. Dryopteris, and D.subg. Erythrovariae) (Wu et al. 2013).

During 2016–2021, we surveyed ferns in the Wulingshan Mountains, which occupy the border zone of four Provinces in China (Hubei, Chongqing, Guizhou and Hunan). This region, as one of the biodiversity hotspots, nurtures a large number of endemic plants and preserves many relict plants (Chen et al. 2004; Yan and Zhou 2021). When we arrived at the Pangu Peak of Dehang Scenic Area, Jishou City, Hunan, an epipetric species that grows in the limestone crevices caught our attention. It is most similar to *Dryopterisgymnophylla* (Baker) C. Chr. and *Dryopterisjishouensis* G.X. Chen & D.G. Zhang but differs by the length to width ratio of lamina, stalks of the basal pinnae, apex form of pinnules and habitat. Moreover, we found this unknown species was also distributed in Mt. Tianmenshan and Zhongli Grand Canyon of Zhangjiajie City, Hunan, China. In order to infer the phylogenetic position of this species, the chloroplast *rbc*L sequences of 32 individuals, representing 11 closely related species, were analyzed. Based on morphological and molecular phylogenetic evidence, we describe it as a new species, named *Dryopteriswulingshanensis* J.P. Shu, Y.H. Yan & R.J. Wang and illustrate it here.

## Materials and methods

### DNA extraction and sequencing

A total of 32 samples, representing 11 species of the genus *Dryopteris*, were analyzed to infer the phylogenetic relationships amongst the unknown species and its closest relatives. *Dryopterisaemula* (Aiton) Kuntze was sampled as an outgroup based on the previous phylogenetic studies of the genus *Dryopteris* (Zhang and Zhang 2012; Zhang et al. 2012). The *rbc*L gene of 18 individuals were newly sequenced and submitted on the GenBank (Table 1), and the others were obtained from GenBank database. Total genomic DNA was extracted from silica gel-dried leaves by using a DNA secure Plant Kit (Tiangen Biotech, Beijing, China) according to the manufacturer’s protocols. The primers and amplification reaction of *rbc*L gene followed the protocols of Shu et al. (2017). Sequencing reactions were set up to obtain both the forward and reverse sequences, and then sequenced on an ABI 3730xl DNA Analyzer (Applied Biosystems, Foster City, California, USA).

**Table 1.** Information of 18 samples newly sequenced in this study.

Taxon	Voucher specimen	Locality	*rbc*L
*Dryopterisjishouensis*	JSL3612	Guangxi, China	MZ444597
	JSL3610	Guangxi, China	MZ444596
	JSL3607	Guangxi, China	MZ444595
	ZXL6320	Hunan, China	MZ444598
	ZXL6317	Hunan, China	MZ444593
	YYH7842	Guizhou, China	MZ444594
*Dryopteriswulingshanensis*	ZXL6320-3	Hunan, China	MZ444607
	ZXL6320-1	Hunan, China	MZ444606
	JSL3935	Hunan, China	MZ444605
	JSL3926	Hunan, China	MZ444604
*Dryopterisgymnophylla*	ZXL6626	Jiangxi, China	MZ444602
	JSL4354	Zhejiang, China	MZ444599
	JSL3320	Anhui, China	MZ444600
	JSL3393	Anhui, China	MZ444601
	JSL3949	Anhui, China	MZ444603
*Dryopterischinensis*	JSL3359	Anhui, China	MZ444610
	JSL3329	Anhui, China	MZ444609
	JSL2983	Anhui, China	MZ444608

### Molecular phylogenetic analysis

The consensus sequences were generated using SeqMan v7.1.0 (DNASTAR, USA) and then 32 sequences used for phylogenetic analysis were aligned with BioEdit v7.2.0 (Hall 1999). The Maximum Likelihood (ML) phylogenetic tree was constructed by IQ-TREE v2.1.3 (Minh et al. 2020), the best-fit model (K2P+I) was chosen according to Bayesian Information Criterion (BIC) with ModelFinder (Kalyaanamoorthy et al. 2017) and the branch support values of ultrafast bootstrap (UFBoot) approximation was performed with 1000 repetitions. Each bootstrap tree was optimized using a hill-climbing Nearest Neighbor Interchange (NNI) search, based directly on the corresponding bootstrap alignment to reduce the risk of overestimating branch supports with UFBoot (Hoang et al. 2017). The Bayesian Inference (BI) species tree was constructed by MrBayes v3.2.7 (Ronquist et al. 2012) with the GTR + I + G model. Four Markov Chain Monte Carlo (MCMC) chains were run simultaneously for two million generations, and sampled every 100 generations. The convergence was assessed with the average standard deviation of split frequencies lower than 0.01.

## Results and discussion

**Figure 1.**
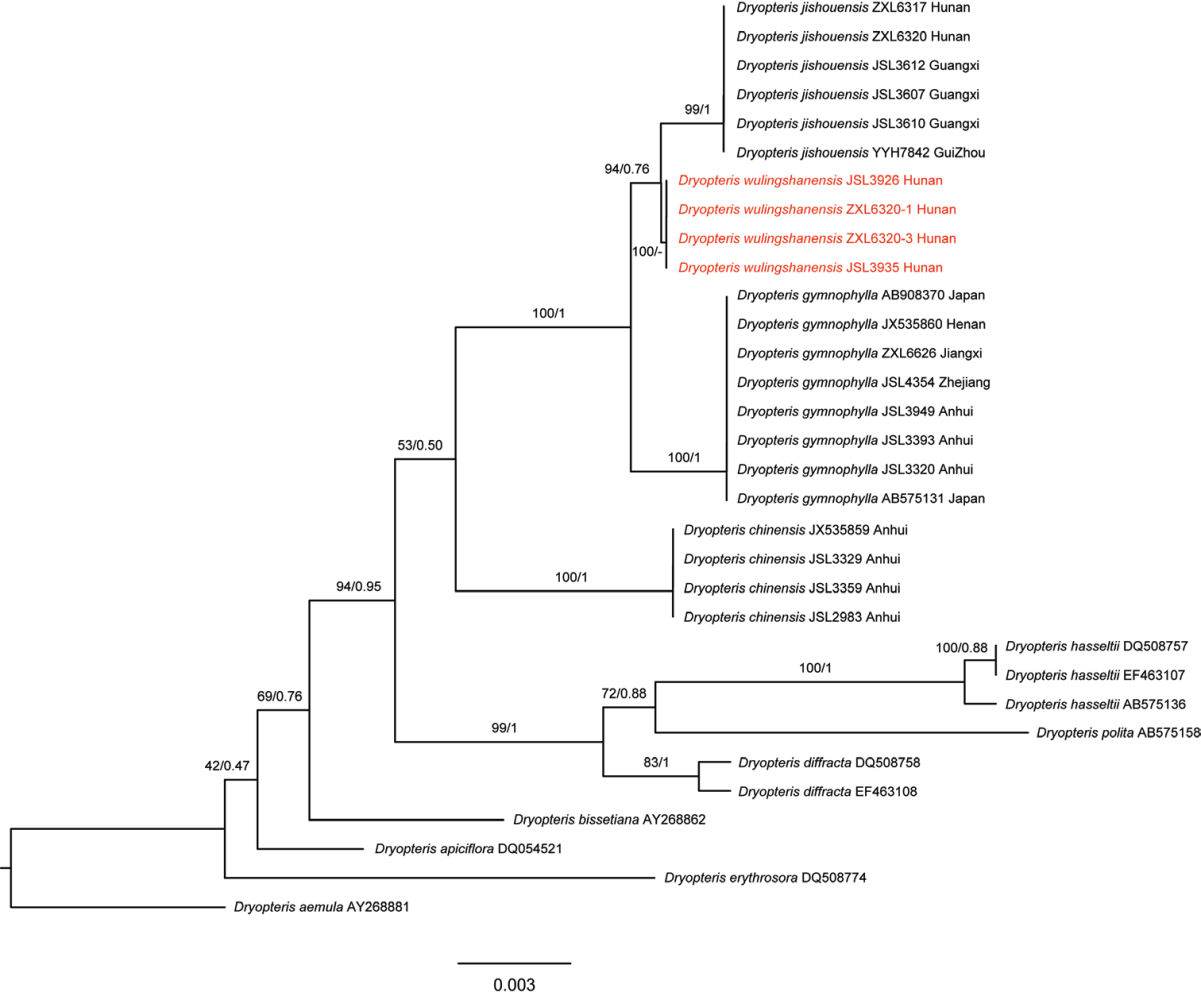
The phylogenetic relationships amongst the new species *Dryopteriswulingshanensis* and its relatives. The topology was the Maximum Likelihood (ML) tree and bootstraps support values (BS) and posterior probability (PP) are showed on the branches.

**Figure 2.**
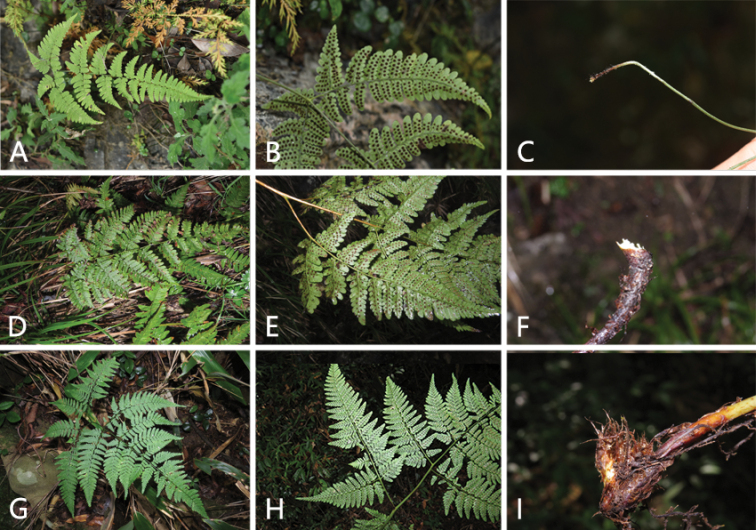
Morphological comparison amongst *Dryopteriswulingshanensis*, *D.jishouensis* and *D.gymnophylla***A–C***D.jishouensis* (type locality, Hunan **A** plants and habitats **B** stalks of basal pinnae **C** scales at base of stipe) **D–F***D.wulingshanensis* (Zhangjiajie, Hunan **D** plants and habitats **E** stalks of basal pinnae **F** scales at base of stipe) **G–I***D.gymnophylla* (Zhejiang **G** plants and habitats **H** stalks of basal pinnae **I** scales at base of stipe).

A total of 32 samples were used for phylogenetic analysis, based on chloroplast *rbc*L gene and the length of sequence alignments was 1,204 bp after removing the missing or gap sites. The phylogenetic relationships amongst *D.wulingshanensis* and its relatives based on ML and BI algorithms, showed a tree topology to be nearly the same as previous studies (Zhang and Zhang 2012; Zhang et al. 2012). Our results indicated the new species *D.wulingshanensis* was a monophyletic clade (BS/PP = 100/-) and was sister to *D.jishouensis* (Fig. 1). Morphologically, the new species was similar to *D.jishouensis* and *D.gymnophylla* on the general morphological characteristics, but differs by the vascular bundles at the base of petiole, length/width ratio of the fronds, stalk length of basal pinnae, division of the lamina, pinnules and habitat (Fig. 2, Table 2).

**Table 2.** The morphological comparison of *Dryopteriswulingshanensis*, *D.jishouensis* and *D.gymnophylla*.

Species characters	*D.jishouensis*	*D.wulingshanensis*	*D.gymnophylla*
Vascular bundles at the base of petiole	2–3	About 5	7–8
Length/width of the fronds	1.7–2.0 times	1.3–1.6 times	1–1.2 times
Stalk of basal pinnae	Usually shorter than 1.5 cm	With a long stalk, usually up to 3cm or more	With a long stalk, usually up to 3cm or more
Division of the lamina	3× pinnate	4× pinnate- pinnatipartite	3× pinnate-pinnatipartite
Pinnules	Obtuse	Acuminate	Acuminate
Habitat	Epipetric	Epipetric	Terrestrial

### Taxonomic treatment

#### Dryopteris wulingshanensis

Taxon classificationPlantaePolypodialesDryopteridaceae

J.P.Shu, Y.H.Yan & R.J.Wang sp. nov.

1E3950D4-1F5B-5F19-82F2-6308056224C2

urn:lsid:ipni.org:names:77222479-1

Figs 3 , 4 武陵山鳞毛蕨

##### Type.

China. Hunan: Wulingshan Mountains Zhongli Grand Canyon, Sangzhi County, Zhangjiajie City. 29°39'10.08"N, 110°37'04.29"E, 900 m alt., 26 June 2021, *Y.-H. Yan & Z.-R. Gu*, *YYH24468* (holotype, IBSC; isotypes, NOCC!, CSH!,PE!).

##### Diagnosis.

The morphology of *D.wulingshanensis* was intermediated between *D.jishouensis* and *D.gymnophylla*, but more similar to the former. *Dryopteriswulingshanensis* and *D.jishouensis* both grow in alkaline soil, but the former’s fronds are ovate, length/width 1.3–1.6 times, tripinnate to quadripartite; the latter’s fronds are ovate-lanceolate to triangular-lanceolate, length/width 1.7–2.0 times or more, bipinnate to tripinnatisect. *Dryopterisgymnophylla* grows in acidic soil, the fronds are pentagonal, usually length/width 1–1.2 times, tripinnate to quadripinnnatisect (Fig. 2, Table 2).

**Figure 3.**
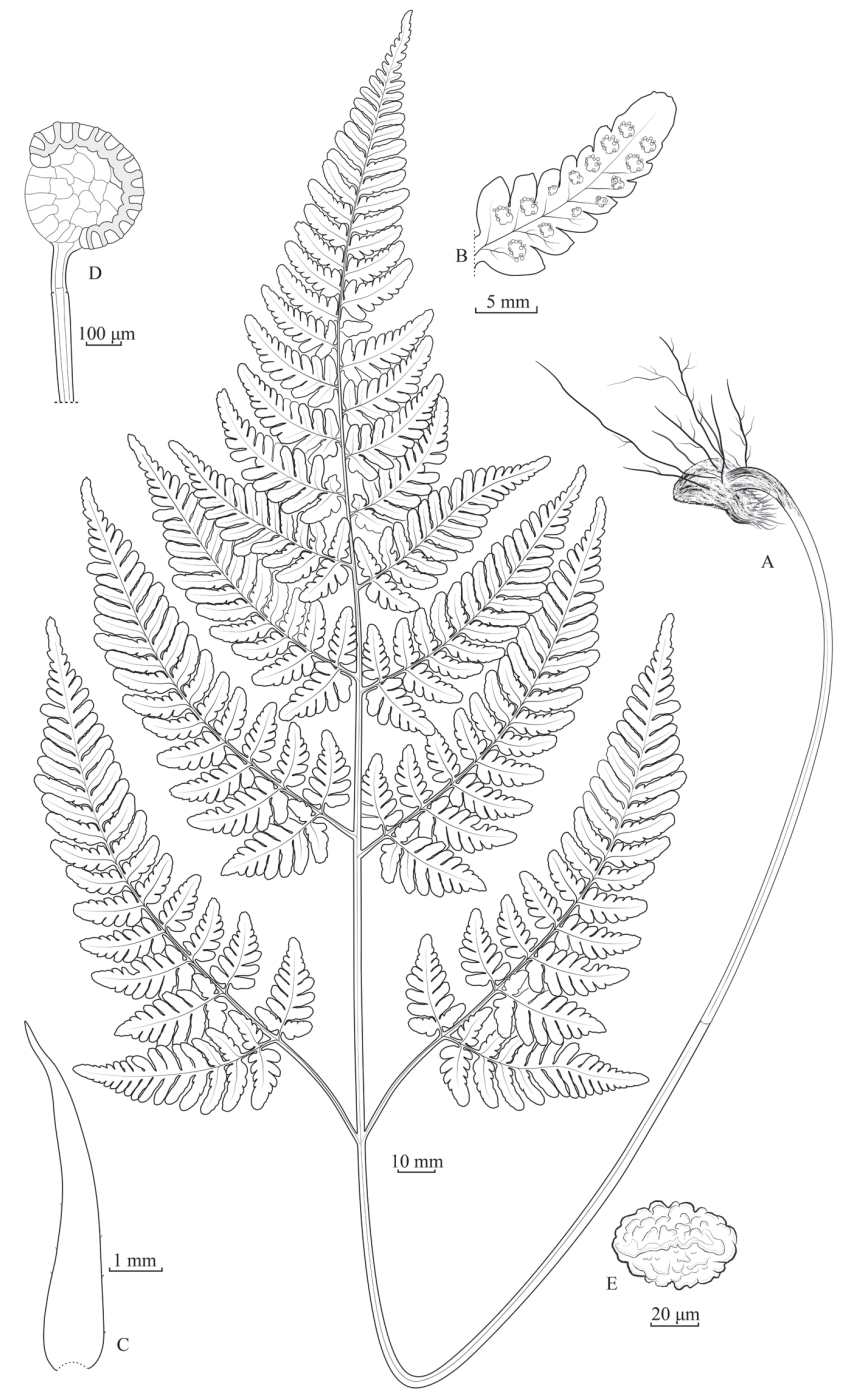
*Dryopteriswulingshanensis* J.P. Shu, Y.H. Yan & R.J. Wang **A** habit **B** pinnule with sori **C** scale at base of stipe **D** sporangium **E** spore (drawn by Li-Jun Chen, based on the type material at IBSC).

**Figure 4.**
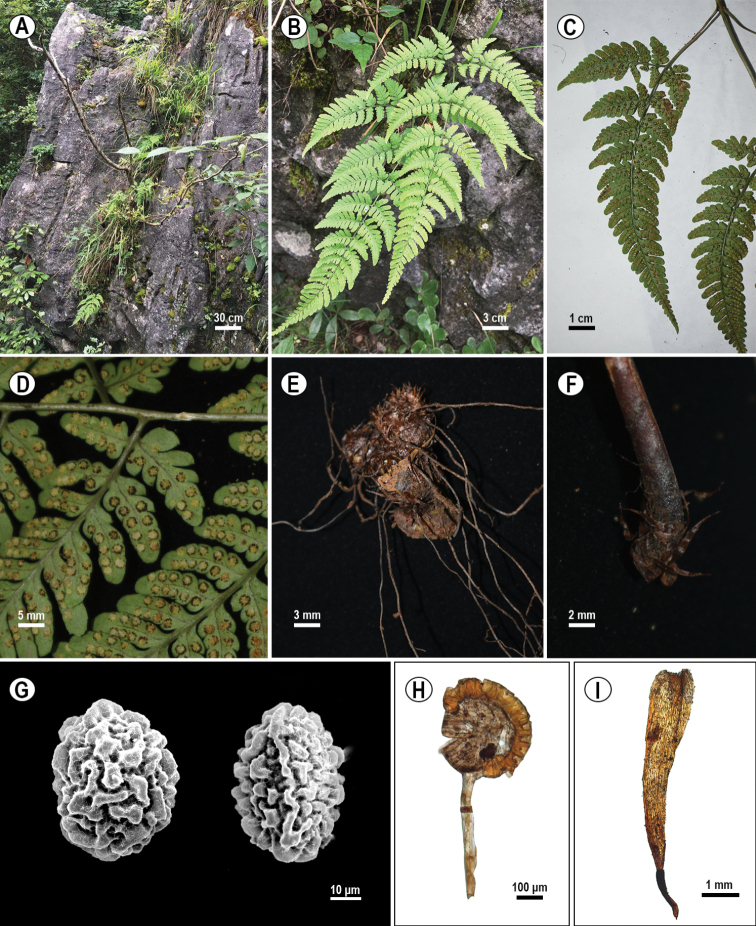
The habitat and morphological characters of *Dryopteriswulingshanensis* J.P. Shu, Y.H. Yan & R.J. Wang **A** habitat **B** lamina **C** basal pinna **D** sori **E** rhizome **F** base of stipe **G** spore (**Left** distal pole, **Right** proximal pole) **H** sporangium **I** scale.

##### Description.

**Rhizome** short-creeping, apex scaly; **scales** dark brown, lanceolate, margin entire or 1–2 dentate at base. **Frond** approximate, (40–)65–70 cm; stipe (23–)31–36 cm, medial diameter 1.5–2 mm, basal scales similar to rhizome scales, antrorse, glabrous, stramineous to brown-stramineous, ventrally grooved; **lamina** ovate, (19–)32–36 × (12–)19–28 cm, about 1.3–1.6 times as long as wide, base round or cordate, apex acuminate, tripinnate to quadripartite (premature lamina only bipinnate to tripinnatisect); **pinnae** 6–8 pairs, oblique, distant, falcate, stipitate, basal pair longest and largest, opposite to nearly opposite, significantly falcate, stipe (1.2–)3–4.5(–7) cm, deltate-lanceolate, (8–)12–23 × (3.6–)7–11 cm, apex long-acuminate, base broad-cuneate to round, asymmetric, basiscopic pinnules longer than acroscopic pinnules, suprabasal pinnae with pinnules similar; **pinnules** 7–8 pairs, discrete, oblique to explanate, falcate, base broadly cuneate, pedicellate, basiscopic ones largest, (3–)5–10 × (1.5–)2.2–4 cm, stipe (1.5–)4–9 mm, trigonal oblong, apex obtuse, ultimate pinnules (lobe) 7–10 pairs, oblique to explanate, trigonal oblong, basiscopic ones longer than acroscopic ones, exstipitate, round-obtuse, base broad-cuneate, connate, pinnatifid to partite, (9–)14–20 (4)8–10 mm; **lobes** 2–7 pairs, 1.5–2 mm wide, oblong, entire, round, others ultimate pinnules lobate to pinnatifid or crenate, base broad-cuneate to decurrent; other pinnae decrescent, opposite to alternate, trigonal lanceolate to oblong lanceolate; **rachis** and costa glabrous, stramineous, adaxially sulcate, lamina papyraceous, glabrous; **veins** pinnate, single or dichotomous; **sori** round, on apices of veinlets, nearly incision; **indusia** orbicular-reniform, margin premorse, brown, persistent.

##### Additional specimens examined (paratypes).

China. Hunan: Pangu Peak, Dehang Scenic Area, Jishou City, Xiangxi Tujia and Miao Autonomous Prefecture. 28°20'50.046"N, 109°34'34.522"E, 914 m alt., 26 Jun 2016, *X.-L. Zhou* et al. *ZXL6320*-*1*, *ZXL6320-3* (CSH!); Tianmenshan Scenic Area, Zhangjiajie City. 5 April 2016, *H.-J. Wei* et al. *JSL3926*, *JSL3935* (CSH!).

##### Distribution and habitat.

*Dryopteriswulingshanensis* is endemic to the Wulingshan mountains in Jishou and Zhangjiajie Cities, Hunan, China. It is epipetric in limestone crevices at an elevation of 700–1000 m in evergreen and deciduous broad-leaved mixed forest. The associated ferns include: *Cheilanthespatula* Baker, *Cyrtomiumfortunei* J. Sm., *Cyrtomiumnephrolepioides* (Christ) Copel., *Lemmaphyllumdrymoglossoides* (Baker) Ching, *Polystichumtsus-simense* (Hook.) J. Sm., *Pronephriumpenangianum* (Hook.) Holttum, *Pyrrosiapetiolosa* (Christ) Ching and *Woodwardiaunigemmata* (Makino) Nakai and associated seed plants include: *Celtissinensis* Pers., *Choerospondiasaxillaris* (Roxb.) B.L. Burtt & A.W. Hill, *Davidiainvolucrata* Baill., Ficussarmentosavar. henryi (King ex Oliv.) Corner, *Hydrangeastrigosa* Rehder, *Loropetalumchinense* (R. Br.) Oliv., *Mallotusphilippinensis* (Lam.) Muell. Arg., *Miscanthussinensis* Andersson, *Platycaryastrobilacea* Siebold & Zucc., *Rhuschinensis* Mill. and *Viburnumcylindricum* Buch.–Ham. ex D. Don.

##### Etymology.

The specific epithet “*wulingshanensis*” is derived from the name of type locality Wulingshan Mountains, where the new species is found.

##### IUCN Conservation Assessment.

EN(B1ab(iii)). *Dryopteriswulingshanensis* is only known from three locations of Wulingshan Mountains in Jishou and Zhangjiajie Cities, Hunan, China. Based on its restricted geographic range, small populations and few individuals, *Dryopteriswulingshanensis* should be considered endangered under the IUCN Red List criteria (IUCN 2019).

## Supplementary Material

XML Treatment for Dryopteris wulingshanensis
